# Supplementary material for: A Review on Recent Approaches on Molecular Docking Studies of Novel Compounds Targeting Acetylcholinesterase in Alzheimer Disease
Source: Molecules. 2023 Jan 21;28(3):1084. doi: 10.3390/molecules28031084 (PMC9921523; doi:10.3390/molecules28031084)
Supplement: Supplementary file 1 [file molecules-28-01084-s001.zip › molecules-2100680-supplementary.pdf]

Table S1: Compounds, reference compounds, inhibitory values and docking results [21, 25, 30-32, 35-38, 40, 42, 46, 48, 50, 62, 68, 70-71, 76-78, 82-83, 87, 92].

Compounds	Reference compounds	IC_{50} (compounds)	IC_{50} (reference compounds)	Lab Test used	Studied Binding Site
**1**	Donepezil	0.451 ± 0.012 μM	0.711 ± 0.017 μM	Healthy male Balb c mice	PAS & CAS
**2**	Donepezil	0.466 ± 0.016 μM	0.711 ± 0.017 μM	Healthy male Balb c mice	PAS & CAS
**3**	Donepezil	0.500 ± 0.018 μM	0.711 ± 0.017 μM	Healthy male Balb c mice	PAS & CAS
**4**	Donepezil	0.590 ± 0.018 μM	0.711 ± 0.017 μM	Healthy male Balb c mice	PAS & CAS
**5**	Galantamine	4.41± 0.53 μg/mL	6.27±1.15 μg/mL	Electrophorus electricus	PAS & CAS
**6**	Galantamine	5.04 ±0.96 μg/mL	6.27±1.15 μg/mL	Electrophorus electricus	PAS & CAS
**7**	Galantamine	0.420 ± 0.019 μM	1.142 ± 0.027 μM	Electrophorus electricus	PAS & CAS
**8**	-	22 ± 2.8 μM	-	Electrophorus electricus	PAS & CAS
**9**	-	31.2 ± 1.8 μM	-	Electrophorus electricus	PAS & CAS
**10**	-	32 ± 2.4 μM	-	Electrophorus electricus	PAS & CAS
**11**	-	36.9 ± 5.6 μM	-	Electrophorus electricus	PAS & CAS
**12**	-	37.6 ± 0.75 μM	-	Electrophorus electricus	PAS & CAS
**13**	Tacrine	0.590 μM	0.71 μM	Electrophorus electricus	PAS & CAS
**14**	-	1.66 ± 0.03 μM	-	Electrophorus electricus	PAS & CAS
**15**	-	pIC50 8.092	-	-	PAS & CAS
**16**	Donepezil	2.95 ± 1.31 μM	0.079 ± 0.05 μM	Electrophorus electricus	PAS & CAS
**17**	-	33.30 μM	-	-	PAS & CAS
**18**	Eserine	128.22± 0.17 μM	0.04 ± 0.0001 μM	Electrophorus electricus	PAS & CAS
**19**	Eserine	16.43±0.48 μM	0.19 ± 0.05μM	Electrophorus electricus	PAS & CAS

**20**	Eserine	43.36 ±0.29μM	0.19 ± 0.05μM	Electrophorus electricus	PAS & CAS
**21**	Eserine	11.73±0.49 μM	0.19 ± 0.05μM	Electrophorus electricus	PAS & CAS
**22**	tacrine	2.75 μM	0.03 μM	Swiss Webster mice	PAS
**23**	Eserine	5.41 ± 0.24 μM	0.19 ± 0.05μM	hAChE (HUMAN)	PAS & CAS
**24**	Eserine	14.29± 0.29 μM	0.19 ± 0.05μM	hAChE (HUMAN)	PAS & CAS
**25**	Eserine	26.75 ± 0.27 μM	0.19 ± 0.05μM	hAChE (HUMAN)	PAS & CAS
**26**	Eserine	13.47 ± 0.31 μM	0.19 ± 0.05μM	hAChE (HUMAN)	PAS & CAS
**27**	Rivastigmine	54.2 ± 1.7 %	59.6 ± 2.6 %	Electrophorus electricus	PAS
**28**	Donepezilb, Galanthamine	4.30 ± 0.3 μM	Don: 0.041 μM<br>Gal: 1.65 μM	Electrophorus electricus	PAS & CAS
**29**	Donepezilb, Galanthamine	3.89 ± 0.3 μM	Don: 0.041 μM<br>Gal: 1.65 μM	Electrophorus electricus	PAS & CAS
**30**	Donepezilb, Galanthamine	2.24 ± 0.4 μM	Don: 0.041 μM<br>Gal: 1.65 μM	Electrophorus electricus	PAS & CAS
**31**	Tacrine	0.099±0.008 μM	0.038±0.007 μM	Electrophorus electricus	PAS & CAS
**32**	Tacrine	506.62 nM	1116.74 nM	Electrophorus electricus	PAS & CAS
**33**	Tacrine	397.32 nM	938.63 nM	Electrophorus electricus	PAS & CAS
**34**	Donepezil	0.044 ± 0.002 μM	0.052± 0.004 μM	Electrophorus electricus	PAS & CAS
**35**	Donepezil	7.58 ± 0.88 μM	0.052± 0.004 μM	Electrophorus electricus	PAS & CAS
**36**	Donepezil	4.10 ± 0.04 μM	0.052± 0.004 μM	Electrophorus electricus	PAS & CAS
**37**	Donepezil	9.71 ± 0.20 μM	0.052± 0.004 μM	Electrophorus electricus	PAS & CAS
**38**	Donepezil	2.70 ± 0.19 μM	0.052± 0.004 μM	Electrophorus electricus	PAS & CAS

**39**	Tacrine, Donepezil	4.17 ± 0.36 µM	Tac: 0.30 ± 0.02 µM-Don: 0.033 ± 0.002µM	Recombinant human AChE	PAS & CAS
**40**	Tacrine, Donepezil	0.73 ± 0.05 µM	Tac: 0.30 ± 0.02 µM-Don: 0.033 ± 0.002µM	Recombinant human AChE	PAS & CAS
**41**	Galantamine	0.86 ± 0.03 µM	1.28 ± 0.01 µM	-	PAS & CAS
**42**	Donepezil	1.98 ± 0.004 µM	0.59 ± 0.083 µM	Electrophorus electricus	PAS & CAS
**43**	Donepezil	3.53 ± 0.42 µM	0.59 ± 0.083 µM	Electrophorus electricus	PAS & CAS
**44**	Donepezil	1.89 ± 0.28 µM	0.59 ± 0.083 µM	Electrophorus electricus	PAS & CAS
**45**	Donepezil	5.58 ± 0.72 µM	0.59 ± 0.083 µM	Electrophorus electricus	PAS & CAS
**46**	Tacrine, Galanthamine	0.74 ± 0.5 µM	Tac: 0.11 ± 0.01 µM Gal: 0.59 ± 0.13 µM	HuAChE	PAS
**47**	Tacrine, Galanthamine	1.31 ± 0.2 µM	Tac: 0.11 ± 0.01 µM Gal: 0.59 ± 0.13 µM	HuAChE	PAS
**48**	Tacrine, Galanthamine	0.077 ± 0.01 µM	Tac: 0.11 ± 0.01 µM Gal: 0.59 ± 0.13 µM	HuAChE	PAS
**49**	Tacrine	14.142 nM	15.0652 nM	Electrophorus electricus	PAS
**50**	Galantamine	0.475 ± 0.12 µM	1.91 ± 0.11 µM	Electrophorus electricus	PAS
**51**	Galantamine	0.547 ± 0.34 µM	1.91 ± 0.11 µM	Electrophorus electricus	PAS
